# A single resistance exercise session improves myocardial contractility in spontaneously hypertensive rats

**DOI:** 10.1590/1414-431X20154355

**Published:** 2015-07-10

**Authors:** A.A. Fernandes, T. de O. Faria, R.F. Ribeiro, G.P. Costa, B. Marchezini, E.A. Silveira, J.K. Angeli, I. Stefanon, D.V. Vassallo, J.H. Lizardo

**Affiliations:** 1 Departamento de Morfologia, Universidade Federal do Espírito Santo, Vitória, ES, Brasil; 2 Departamento de Ciências Fisiológicas, Universidade Federal do Espírito Santo, Vitória, ES, Brasil; 3 Departamento de Ciências Fisiológicas, Escola Superior de Ciências da Santa Casa de Misericórdia de Vitória, Vitória, ES, Brasil

**Keywords:** Cardiac, Contractility, Myocardium, Proteins, Resistance exercise

## Abstract

Resistance training evokes myocardial adaptation; however, the effects of a single resistance exercise session on cardiac performance are poorly understood or investigated. This study aimed to investigate the effects of a single resistance exercise session on the myocardial contractility of spontaneously hypertensive rats (SHRs). Male 3-month-old SHRs were divided into two groups: control (Ct) and exercise (Ex). Control animals were submitted to sham exercise. Blood pressure was measured in conscious rats before the exercise session to confirm the presence of arterial hypertension. Ten minutes after the exercise session, the animals were anesthetized and killed, and the hearts were removed. Cardiac contractility was evaluated in the whole heart by the Langendorff technique and by isometric contractions of isolated left ventricular papillary muscles. SERCA2a, phospholamban (PLB), and phosphorylated PLB expression were investigated by Western blot. Exercise increased force development of isolated papillary muscles (Ex=1.0±0.1 g/mg *vs* Ct=0.63±0.2 g/mg, P<0.05). Post-rest contraction was greater in the exercised animals (Ex=4.1±0.4% *vs* Ct=1.7±0.2%, P<0.05). Papillary muscles of exercised animals developed greater force under increasing isoproterenol concentrations (P<0.05). In the isolated heart, exercise increased left ventricular isovolumetric systolic pressure (LVISP; Δ +39 mmHg; P<0.05) from baseline conditions. Hearts from the exercised rats presented a greater response to increasing diastolic pressure. Positive inotropic intervention to calcium and isoproterenol resulted in greater LVISP in exercised animals (P<0.05). The results demonstrated that a single resistance exercise session improved myocardial contractility in SHRs.

## Introduction

Arterial hypertension (AH) is associated with a higher risk of cardiac abnormalities due to hypertrophy of myocardial cells and capillaries, and of interstitium abnormalities that lead to alterations in myocardial structural and molecular mechanisms, resulting in development of cardiomyopathy and heart failure (1). Although treatment of hypertension involves several therapeutic procedures, nonpharmacological treatment of AH consists of reducing body weight, adhering to a low-sodium diet rich in fruits and vegetables, quitting smoking, eliminating alcohol consumption, and doing regular physical exercise (2).

Isotonic exercise associated with resistance exercise is capable of decreasing resting blood pressure (BP) and is indicated not only for treatment of hypertension but also for prevention of hypertension (3). Previous studies have reported enhanced cardiac function as a result of endurance training ([Bibr B04] [Bibr B05] 46). Endurance training improves cardiomyocyte contractility and calcium (Ca^2+^) handling and increases isometric tension development by the myocardium, resulting in cardiac performance optimization (7). De Cássia et al. (8) showed that the increase in force development by isolated papillary muscles, after resistance training, was associated with an increase in myosin ATPase activity.

Recently, attention has been given not only to the beneficial cardiovascular effects of physical training but also to the effects resulting from a single exercise session (9). It is well established that a single resistance exercise session decreases resting BP and improves vascular function in humans and hypertensive animals (10,11). Nevertheless, the effects of a single resistance exercise session on myocardium contractility are still unknown. Thus this study was designed to investigate the effects of a single resistance exercise session on the myocardial contractility of spontaneously hypertensive rats (SHRs).

## Material and Methods

### Animals

The experiments were conducted using 3-month-old male SHRs that weighed 250-300 g. The rats were housed in cages with controlled room temperature, humidity, and light cycles (12:12-h light-dark cycle); the animals had free access to tap water and were fed a standard rat chow *ad libitum*. Care and use of the laboratory animals and all experiments were conducted in accordance with the United States National Institutes of Health *Guide for the Care and Use of Laboratory Animals*, and the protocols were approved by the Ethics Committee of the Escola Superior de Ciências da Santa Casa de Misericórdia de Vitória, Vitória, ES, Brazil (CEUA-EMESCAM, No. 009/2007).

### Surgical procedures

All surgical procedures were performed using aseptic techniques. Anesthesia was induced with intraperitoneal (*ip*) injections of ketamine (50 mg/kg) and xylazine (10 mg/kg), and supplementary doses were administered if the rats regained a blink reflex. The left carotid artery was carefully isolated to avoid damage to any nearby nerves. A tapered polyethylene cannula (PE50) that was filled with heparinized saline (100 units/mL) was inserted into the left common carotid artery to measure BP. The free end of a catheter was plugged with a stainless steel obturator and was inserted subcutaneously to exit from the back of the neck. The animals were placed in separate cages and were allowed to recover for 24 h before initiating the experimental procedures. The rats were monitored for any signs of infection.

BP and heart rate were continuously recorded in conscious rats before the resistance exercise session by connecting the arterial catheter to a TSD104A pressure transducer that was coupled to a DA100C amplifier. An acquisition system (MP 100 Biopac Systems, Inc., USA) was used for real-time BP and heart rate recording and subsequent analysis. This protocol was performed to confirm the presence of AH in the experimental groups.

### Experimental groups

On the day of the experiment, the rats were allowed to adapt to the laboratory environment for 1 h before their resting hemodynamic measurements were recorded. After the adaptation period, baseline BP values were measured in conscious animals for 10 min. Subsequently, the animals were randomly divided into two experimental groups: the exercised group (Ex, n=6), in which rats were submitted to a single resistance exercise session, or the control group (Ct, n=6), in which the animals were only submitted to a single simulation session. Before the exercise session, BP was measured again for 30 min.

### Exercise protocol

Initially, the animals were allowed to adapt to the exercise apparatus for 4-5 days. Afterward, the maximum weight lifted (1RM) with the squat-training apparatus was measured; 1RM was defined as the maximum weight that was lifted by each rat using the exercise apparatus. After 2 days of rest, the animals were submitted to an exercise protocol. The rats performed a single resistance exercise session according to a model that was adapted from Tamaki et al. (12). Rats wearing a canvas jacket were able to regulate the twisting and flexion of their torsos and were fixed by a holder in a standing position on their hind limbs. Electrical stimulation (20 V for 0.3 s duration and at 3-s intervals) was applied to the rat tails through a surface electrode. As a result, the animals extended their legs repeatedly, which lifted the weight on the arm of the exercise apparatus. This apparatus was chosen because it mimics traditional squat exercises performed by humans, and the results obtained in rat skeletal muscles are similar to those observed in humans (12). The rats were exercised for 20 sets with 15 repetitions per set in the exercise apparatus. The repetitions were performed at 3-s intervals with a 1-min rest period between the sets. The exercise intensity was 50% of 1RM. The control group received the same stimulus at the same frequency and duration and at the same intensity and intervals as the Ex group. However, the exercise apparatus was unweighted and in the rest position. Therefore, these animals did not lift a load. Exercised and control animals were randomly allocated in sequence into one of the following groups for experimental measurements: measurement of cardiac contractility by isolated heart perfusion, measurement of myocardial contractility by isolated papillary muscles, and Western blot analysis.

### Isolated heart perfusion

After the BP measurements, rats were anesthetized with urethane (1.2 g/kg), treated with heparin (500 IU, *ip*) and killed by exsanguination. The heart was excised after 10 min, mounted in an isolated organ chamber, and perfused according to the Langendorff technique (13) with a constant flow (10 mL/min) of Krebs Henseleit bicarbonate buffer solution containing the following: 120 mM NaCl, 5.4 mM KCl, 1.25 mM CaCl_2_, 2.5 mM MgSO_4_, 1.2 mM Na_2_SO_4_, 2.0 mM NaH_2_PO_4_, 20 mM NaHCO_3_, and 11 mM glucose (salts used were of analytical grade; Sigma, USA, and Merck, Germany). This solution was filtered, continuously bubbled with 95% O_2_ and 5% CO_2_, pH 7.4, and kept between 34° and 35°C. After the heart was mounted, the left atrium was opened and a soft distensible balloon mounted at the tip of a rigid plastic tube was inserted into the left ventricular cavity through the atrioventricular valve. To avoid liquid accumulation in the ventricular cavity, the ventricle was perforated with a puncture needle.

The balloon was connected, via a Y piece, to a pressure transducer (TSD 104A) and a syringe so that the diastolic pressure of the left ventricle could be adjusted to predetermined values by injecting water into the balloon. The resulting pressure was registered. The hearts were driven with isolated suprathreshold rectangular pulses (5-ms duration) at a constant rate (3.3 Hz) through a pair of Ag/AgCl electrodes attached to the upper region of the right ventricle. Mechanical activity was investigated by measuring the developed left ventricular isovolumetric systolic pressure (LVISP). To evaluate contractility, the rate of LVISP increase (dP/dt) was used because LVISP is highly sensitive to changes in contractility (14). These parameters were measured with a pressure transducer (TSD104A pressure transducer coupled to a DA100C amplifier) connected to a data acquisition system (BIOPAC MP100WSW, including the AcqKnowledge III software, USA). The isovolumetric pressure derivative (dP/dt) was recorded offline by the same software (digital filter Blackman −61 dB, 25 kHz of cut frequency and sample rate of 1000/s). All measurements began 30 min after mounting to allow the beating preparation to adapt to the *in vitro* conditions.

The protocols were performed by adjusting the volume of the balloon, beginning with a constant diastolic pressure of 5 mmHg. Ventricular function curves were obtained by measuring LVISP while diastolic pressure was increased from 0 to 30 mmHg at 5-mmHg intervals. The balloon volume was kept constant during experiments involving other protocols; this permitted changes in diastolic and systolic pressures to be measured. Initially, the recordings were taken under control conditions in both groups. To analyze the inotropic response, a single dose of isoproterenol (Sigma) in bolus (100 μL, 10 μM) was administered to evaluate the β-adrenoceptor response.

### Isolated papillary muscles

Rats received 500 units of heparin, *ip*, and were anesthetized 10 min later with urethane 1.2 g/kg (Sigma). The hearts were rapidly removed and perfused through the aortic stump as the left ventricle papillary muscles were dissected. Muscle preparations were mounted for isometric tension recording and maintained in 20 mL Krebs-Henseleit solution: 118 mM NaCl, 4.7 mM KCl, 1.25 mM CaCl_2_, 1.2 mM KH_2_PO_4_, 1.2 mM MgSO_4_, 23 mM NaHCO_3_, and 11 mM glucose) at 30°C and pH 7.4, which was continuously aerated with 95% O_2_ and 5% CO_2_ ([Bibr B15] [Bibr B16] 1517). Resting tension was adjusted to produce maximal contractile force. The twitch contraction rate was controlled by isolated rectangular pulses (10-15 V, 12-ms duration) through a pair of platinum electrodes. The standard stimulation rate was 0.5 Hz (steady state). Isometric force development was measured with an isometric force transducer (TSD105A, Biopac) and normalized to muscle weight (g/g). Recording started after 30 min to allow the muscle to adapt to the new environmental conditions. Myocardial contractility was tested by measuring the positive inotropic response to isoproterenol (Sigma) concentrations to the bath (5×10^−7^ to 5×10^−2^).

Post-rest potentiation (PRP) was used to provide indirect information about the function of the sarcoplasmic reticulum (SR). PRP depends on the pause duration and on the amount of calcium stored at intracellular sites. Pause intervals of various durations (15, 30, and 60 s) were used, and the results are reported as relative potentiation (the amplitude of PRP divided by steady-state contractions) to normalize the data from different preparations. Post-rest contraction (PRC) was obtained after 10 min without stimulation and in the calcium-free solution containing 5 mM caffeine. To achieve PRC, the calcium-free solution was replaced with Krebs’s solution (with 1.25 mM calcium) seconds before the electric stimulation. The first contraction after rest was taken as an index of the sarcolemmal calcium influx.

### Western blot analyses

Western blotting was performed as previously described (18). Proteins from homogenized left ventricles were separated by 10% or 15% SDS-PAGE. Proteins were transferred onto nitrocellulose membranes, which were incubated with mouse monoclonal antibodies for SERCA-2a (1:1000, Affinity BioReagents, USA), phospholamban (PLB; 0.5 µg/mL, Affinity BioReagents), and PLB phosphorylated at serine 16 (pPLB^Ser16^ 1:5000, Badrilla, UK). After they were washed, the membranes were incubated with anti-mouse (1:5000, Stressgen, Canada) or anti-rabbit (1:7000, Stressgen) immunoglobulin antibodies conjugated to horseradish peroxidase. After they were thoroughly washed, immunocomplexes were detected using an enhanced horseradish peroxidase/luminol chemiluminescence system (ECL plus, Amersham International, UK) and film (Hyperfilm ECL International). Signals on the immunoblot were quantified with the Image J computer program (NIH, USA). Each membrane was reprobed to determine GAPDH expression using a monoclonal mouse antibody (1:5000, Abcam Cambridge, USA).

### Data analysis and statistics

Data are reported as means±SE. The results were analyzed by one-way and two-way analysis of variance (ANOVA) for repeated measurements and by the unpaired Student's *t*-test. When significant differences were obtained by one-way or two-way ANOVA, *post hoc* analyses were performed using Tukey’s least significant difference test. For protein expression, data are reported as the ratio between the signals on the immunoblot that correspond to the protein of interest and to GAPDH; P<0.05 was considered to be statistically significant.

## Results

Table 1 presents hemodynamic values and maximal load lifted by control and exercised rats. Rats from both groups presented similar values for BP and maximal lifted load (P>0.05).

**Table:**
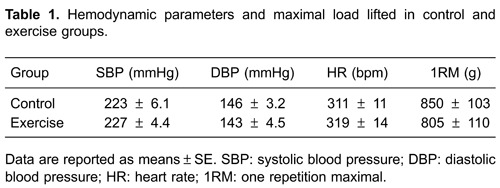


### Isolated heart perfusion

**Figure 1:**
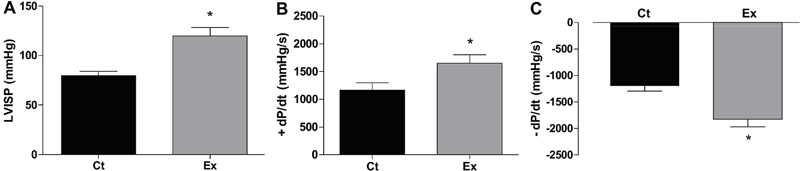
Contractility values from isolated heart. *A*, left ventricular isovolumetric systolic pressure (LVISP); *B*, positive first time derivative (+dP/dt), and *C*, negative first time derivative (−dP/dt) from control (Ct, n=6) and exercised (Ex, n=6) spontaneously hypertensive rats (SHR) under control conditions in Langendorff apparatus. Results are reported as means±SE. *P<0.05, compared to Ct (Student’s *t*-test).

**Figure 2:**
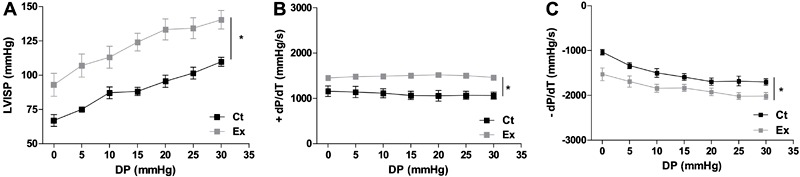
Contractility values from isolated heart. *A*, left ventricular isovolumetric systolic pressure (LVISP); *B*, positive first time derivative (+dP/dt), and *C*, negative first time derivative (−dP/dt) curves obtained at different diastolic pressures (DP) from control (Ct, n=6) and exercised (Ex, n=6) spontaneously hypertensive rats (SHR). Results are reported as means±SE. *P<0.05 (two-way ANOVA).

To investigate cardiac effects, Langendorff-perfused hearts from both control and exercised rats were used. Figure 1 shows that LVISP was increased in the Ex group (Δ+39 mmHg; P<0.05) with diastolic pressure fixed at 5 mmHg. Similarly, positive and negative dP/dt also increased (Δ+487 mmHg/s; Δ−631 mmHg/s; P<0.05). When performing the ventricular function curves, LVISP was increased in the Ex group for all diastolic pressure values (P<0.05; Figure 2A). Similar results were obtained with positive and negative dP/dt (P<0.05; Figure 2, B and C).

**Figure 3:**
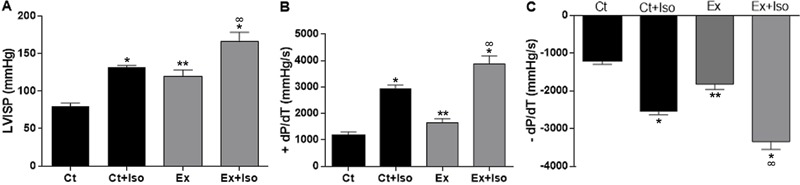
Effect of β-adrenergic activation by isoproterenol [Iso (100 μL, 10 μM, in bolus)] in the Langendorff apparatus on (*A*) left ventricular isovolumetric systolic pressure (LVISP), (*B*) positive first time derivative (+dP/dt), and (*C*) negative first time derivative (−dP/dt) from control (Ct, n=6) and exercised (Ex, n=6) spontaneously hypertensive rats (SHR). Results are reported as means±SE. *P<0.05, *vs* baseline condition; **P<0.05 *vs* Ct, ∞P<0.05 *vs* Ct+Iso (one-way ANOVA).

Isoproterenol was used to test whether acute exercise training could alter the myocardial response to inotropic interventions. Isoproterenol administration (100 μL, 10 μM, in bolus) increased LVISP and positive and negative dP/dt in both groups; these values (presented as Δ) were higher in the Ex group (165.8±12.6 *vs* 132±2.0 mmHg, P<0.05; Ex+Iso: Δ+dP/dt 2228 mmHg/s, Δ−dP/dt 1523 mmHg/s *vs* Ct+Iso: Δ+dP/dt 1786 mmHg/s, Δ−dP/dt 1354 mmHg/s; P<0.05), suggesting an increase in the β-adrenergic inotropic action (Figure 3B and C).

### Isolated papillary muscles

In isolated papillary muscles, a single resistance exercise session increased the isometric force developed (Ex=1.0±0.1 g/mg) in exercised hypertensive rats when compared with the control group (Ct=0.63±0.2 g/mg; P<0.05). To investigate the putative role of the SR and sarcolemmal calcium influx, two protocols were evaluated: PRP and PRC.

**Figure 4:**
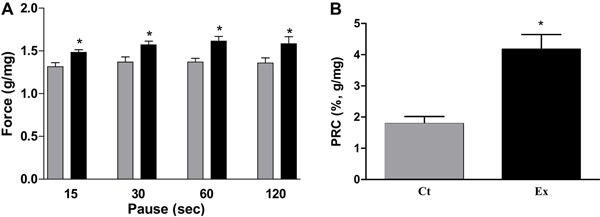
Effects of post-rest potentiation (*A*) and post-rest contraction (PRC) (*B*) on force developed in isolated left ventricle papillary muscles from control (Ct, n=16) and exercised (Ex, n=16) spontaneously hypertensive rats (SHR). Gray bars: control; black bars: exercise. Results are reported as means±SE. *P<0.05, compared to control (Student’s *t*-test).

**Figure 5:**
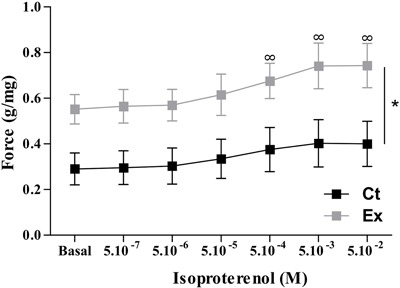
Effects of increasing extracellular β-agonist isoproterenol (5.10^-7^-5.10^-2^ M) on force developed in isolated left ventricle papillary muscles from control (Ct, n=16) and exercised (Ex, n=16) spontaneously hypertensive rats (SHR). Results are reported as means±SE. *P<0.05 Ex *vs* Ct; ^∞^P<0.05 *vs* basal and 5.10^-7^ and 5.10^-6^ (two-way ANOVA).

In the first protocol, after all pauses, the relative contraction force was increased in the Ex group (P<0.05; Figure 4A). In the second protocol, exercise significantly increased the PRC when compared with control rats (Ex=4.1±0.4% *vs* Ct=1.7±0.2% g/mg, P<0.05; Figure 4B). As expected, isoproterenol promoted a positive inotropic effect in all groups examined. Moreover, the papillary muscles of the exercised animals developed greater force under increasing isoproterenol concentrations (P<0.05, Figure 5). To evaluate the putative role of contractile proteins in the increase of force, we performed tetanic contractions, which evoke maximal activation of contractile proteins. No changes in tetanic contractions after exercise (P<0.05) on either the peak or the plateau were observed (data not shown).

### Western blot analyses

**Figure 6:**
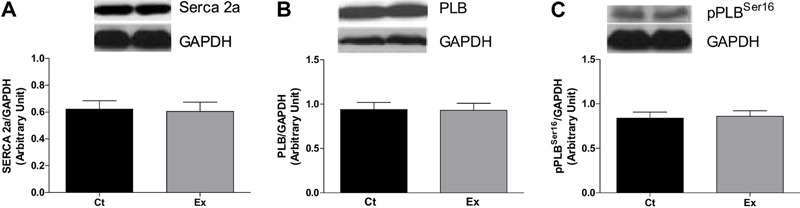
Densitometric analysis of the Western blot for (*A*) SERCA2a, (*B*) phospholamban PLB, and pPLB^Ser16^ (*C*) from controls (Ct, n=6) and exercised (Ex, n=6) spontaneously hypertensive rats (SHR). Results are reported as means±SE. P>0.05 (Student’s *t*-test).

To evaluate the role of regulatory SR proteins, SERCA-2a, PLB, and pPLB^Ser16^ expression were measured by Western blot techniques. No differences in protein expression were observed between the groups (P>0.05, Figure 6).

## Discussion

The present study demonstrated that a single resistance exercise session increased isometric force development of isolated papillary muscles and the cardiac performance of isolated perfused hearts. However, the session did not alter the expression of proteins that regulate functional activity of the SR.

AH imposes a higher pressure overload on the myocardium due to the increase in peripheral vascular resistance, promoting hypertrophy (19). These compensatory responses in the cardiac cell occur to maintain the function of the cardiac pump and decrease ventricular wall tension while pressure values stay high (20).

It is well established that aerobic exercise and resistance training improve ventricular function through cardiac adaptations; ventricular hypertrophy increases cardiac output during exercise and improves diastolic function ([Bibr B21] [Bibr B22] 2123). However, there are no reports of studies that investigated changes in myocardial contractility after a single resistance exercise session in hypertensive rats. Thus the aim of the present investigation was to evaluate myocardial contractility performance in the isolated heart perfused by the Langendorff technique, the force developed by isolated papillary muscles, and SERCA-2A and PLB expression after an acute resistance exercise session in SHRs.

### Contractility of isolated heart by the Langendorff technique

The results obtained showed that a single resistance exercise session increased LVISP as well as +dP/dt and −dP/dt from basal conditions. After inotropic intervention with isoproterenol, exercise increased +dP/dt and −dP/dt. The increase in the pressure developed by the heart reflects the heart's function as a pump. Thus, the increased LVISP shows improved heart performance to increase cardiac output. To evaluate myocardial contractility, which is defined as the inotropic state of the cardiac muscle (24), dP/dt was used; dP/dt is an important index of myocardial contractility because it is directly influenced by cardiac inotropism (25). Our findings suggest that a single acute resistance exercise session was capable of increasing cardiac pump performance. Previous studies have demonstrated that cardiac function of isolated hearts is enhanced after aerobic training (26). Using an apparatus similar to that used in the present study, Penpargkul and Scheuer (26) found greater cardiac output and dP/dt in the isolated hearts of Wistar rats after swimming. Moreover, this study demonstrated a higher contractile response (LVISP and dP/dt) due to the increased left atrium filling pressure in exercised rats.

In the isolated heart, the Frank-Starling mechanism was also evaluated by a stepwise increase of the diastolic pressure. For all diastolic pressures, LVISP was higher in exercised animals. Previous studies using isolated hearts of hypertensive and normotensive rats have demonstrated similar results ([Bibr B27] [Bibr B28] 2729). MacDonnell et al. (30) showed that exercise training increased the diastolic compliance of SHR hearts and, in turn, resulted in an improvement in the Frank-Starling relationship. Enhanced diastolic compliance might be associated with a lower intracellular calcium concentration during diastole, which is influenced by SR calcium reuptake. Under such conditions, enhanced relaxation enables better diastolic filling of the heart, which might increase cardiac output.

### Contractility of left ventricle papillary muscles

A single resistance exercise session evoked a significant increase in the isometric force development of the left ventricle isolated papillary muscles, corroborating the enhanced cardiac performance registered in the isolated heart. Several studies aimed to investigate the effects of exercise training on myocardial contractility (8,); nevertheless, none of those studies investigated the acute effects of exercise. Williams and Potter (31) found that 6 weeks of treadmill training did not alter contractility of the right ventricular papillary muscles of cats. Similarly, Collins et al. (33) showed that aerobic training did not influence contractility of the left ventricle papillary muscles in rats. In addition, Wyatt et al. (34) demonstrated an increase in contractile function of the left ventricle papillary muscles in rats after 20 weeks of swim training. Previous findings from our laboratory (8) using the same exercise apparatus as in the present study also showed improved contractile function of the left ventricle papillary muscles of Wistar rats after eight resistance training sessions. The mechanism involved in improving cardiac contractile function after exercise training may be different from that involved with acute exercise, because training evokes cardiac hypertrophy. Wyatt et al. (34) reported that an increase in the isometric force of the papillary muscles was evident only when the development force was not corrected by the weight of the papillary muscles. In these animals, significant cardiac hypertrophy was observed after training; thus, contractile performance could not be attributed to the enhanced number of contractile units.

In cardiac muscle, the contractions occurring after short pauses are potentiated and are called PRCs (15). The cardiac muscle of rats increases its force as the rest period increases (16). The PRCs depend on pause duration and on the amount of calcium stored in intracellular sites (17). Thus, the relative participation of the SR is important for PRCs, and this parameter can be used as an indicator of SR function. Our results demonstrated that only the exercised rats presented potentiation of contractile force after pauses. These findings suggest that after exercise the SR reuptakes and releases more calcium, resulting in improved contractility, which indicates an increase in SR function. This mechanism could explain the increased performance of the isolated heart and the increase in isometric force development by isolated papillary muscles. In accordance, the findings obtained with the PRP maneuver demonstrated an increase in SR functional activity. It is well established that SR function is regulated by PLB and SERCA-2a activity. Thus, we hypothesized that an upregulation of these proteins may be associated with improved cardiomyocyte contractility; however, we did not observe alterations in PLB or SERCA-2a expression after exercise.

Improved SR functional activity could have also played a role in the increase in LVISP when the Frank-Starling mechanism was elicited, as observed in exercised animals. The stretching of cardiac myocytes during diastole opens stretch-sensitive calcium channels, increasing the availability of these ions for SR reuptake, which might enhance ventricular contraction. In accordance with our findings, Penpargkul et al. (35) showed an increase in calcium transport by the SR in Wistar rats after 8 weeks of swim training. Moreover, this study demonstrated that exercise training was capable of increasing the capacity for calcium storage in microsomes prepared from the hearts. Despite the use of acute exercise and no training in the present investigation, our results are in agreement with the findings obtained by Penpargkul et al. (35).

Cardiac contraction is also regulated by the sympathetic nervous system, and β-adrenergic stimulation increases the contraction force and heart rate (36). In the present study, the inotropic response to isoproterenol was potentiated in the isolated heart after acute exercise. It was observed by analysis of dP/dt. These findings are in accordance with previous findings in humans and animals (8,30,34,). Interestingly, MacDonnell et al. (30) showed in SHRs that aerobic training improves the inotropic and lusitropic responsiveness to β-adrenergic receptor stimulation and increases PLB phosphorylation. Consequently, calcium reuptake and subsequent release by the SR increase. Another response that could explain the increased isoproterenol response is the increase in cardiac myosin adenosine triphosphatase activity (8) and the improvement in calcium influx by the sarcolemma. We indirectly tested this influx using PRC that depends on calcium influx (15). The results showed an increase in PRC, suggesting an increase in sarcolemmal calcium influx, which helps to explain the improved isoproterenol response in the isolated heart and the isolated papillary muscles. Cardiac myosin ATPase activity was not examined in the present study, although we investigated the influence of exercise on contractile proteins using tetanic contractions. Tetanic contractions are obtained after inhibition of SR activity with caffeine. Caffeine acts by depleting the SR of its calcium content and also by inhibiting Ca^2+^ reuptake. This maneuver has been used to produce maximal activation of the contractile machinery in the intact myocardium (40). No changes were observed in this maneuver after exercise, suggesting that improved contractile performance in the papillary muscles and isolated heart was not mediated by contractile proteins.

In summary, the results obtained in isolated heart and papillary muscles suggest an increase in myocardial contractile performance after a single resistance exercise session. This improvement in contractile activity seems to be mediated by an increase in SR activity and an increase in sarcolemmal calcium influx. Therefore, a single resistance exercise session might improve myocardium contractility in SHRs and might be an important strategy for the prevention and non-pharmacological treatment of cardiac damage evoked by AH in humans.
